# Mechanism of Solid Ammonia Stabilization at Ambient Temperature: Insights from Thermodynamics, Phonon Calculation, and Micromechanics

**DOI:** 10.1021/acsomega.5c09165

**Published:** 2025-11-17

**Authors:** Masao Morishita, Terumasa Tadano, Yusuke Matsuoka, Taichi Abe

**Affiliations:** † Research Center for Structural Materials, 52747 National Institute for Materials Science, 1-2-1 Sengen, Tsukuba, Ibaraki 305-0047, Japan; ‡ Research Center for Magnetic and Spintronic Materials, National Institute for Materials Science, 1-2-1 Sengen, Tsukuba, Ibaraki 305-0047, Japan

## Abstract

The stabilization of low-temperature solid-state ammonia at ambient temperature was recently achieved by confining fine crystals within a boric acid glass matrix through freeze-drying using liquid nitrogen, opening a new frontier for hydrogen storage materials. We determined the stabilization mechanism using a thermodynamic model supported by first-principles phonon calculation and pressure analysis based on micromechanics. Phonon calculation revealed vibrational densities of states (DOS) for the intermolecular translational and rotational modes of NH_3_ molecules, as well as intramolecular librational and bending modes of hydrogen atoms. Simulations incorporating the computed DOS into a thermodynamic model reveal that sub-GPa pressure is exerted on ammonia crystals by the surrounding glass matrix. Micromechanical analysis confirms that this hydrostatic stress originates from the difference in thermal expansion coefficients. The results suggest that ammonia crystals are stabilized by this pressure. Thus, our findings provide a new strategy for stabilizing nonequilibrium phases at ambient temperature, opening new frontiers for hydrogen storage materials.

## Graphical abstract

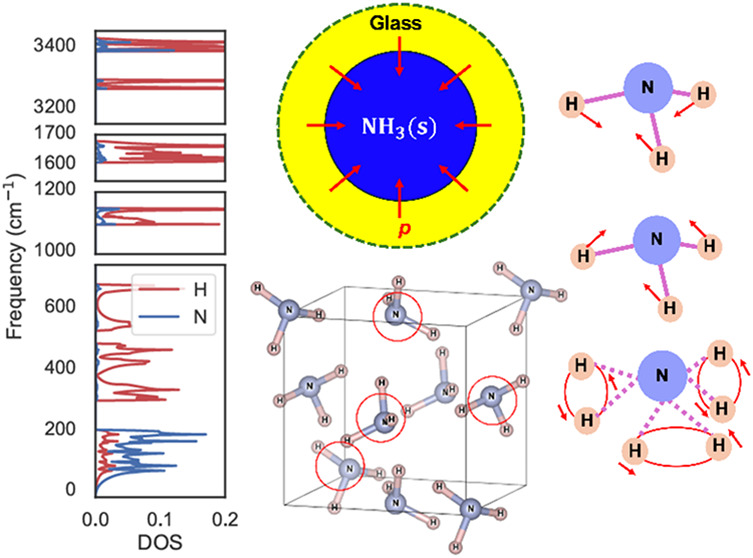

## 1 Introduction

Alternative energy resources such as solar photovoltaic technology and wind power are widely explored to reduce CO_2_ emissions from fossil fuels. However, the consistent supply of these resources is limited because of their reliance on weather and geography limits consistent supply. This has increased the difficulty of meeting growing energy demands and intensified the need for alternative energy storage solutions. One promising alternative is green hydrogen, which is produced by water electrolysis using renewable energy. This hydrogen can be reconverted into electricity using fuel cells or used directly as a combustion fuel. However, the energy density of H_2_(g) is nearly 1/3000th that of gasoline due to its low volumetric density.^1^ Moreover, storing and transporting H_2_(l) is challenging because of its extremely low boiling point (*T*_b.p._^°^ = 20 K) and the high cost of liquefaction needed to increase its energy density.

Ammonia is a promising hydrogen carrier,^2–11^ and when produced from green hydrogen, it is termed green ammonia. At ambient temperature, it can be stored as a liquid in standard cylinders due to its liquefaction at ∼8 atm and 20 °C.^12^ Ammonia is industrially produced via the Haber–Bosch process,^2^ which uses an iron catalyst under high temperature and pressure to combine hydrogen and nitrogen. Additionally, ammonia production has been explored via the cleavage of the strong N–N bonds in N_2_(g) using electrolysis,^3–5^ discharge,^6^ and surface plasmon resonance.^7^ Conversely, ammonia can be converted into H_2_(g) and N_2_(g) using catalysts such as Ni,^8,9^ Ru,^9^ and CaNH,^10^ while the generated H_2_(g) is separated using membranes.^11^ However, the storage and transport of ammonia remain challenging as it is a deleterious substance. Damage to pressurized vessels during transit releases toxic ammonia gas, posing serious risks and even fatalities. In our previous study,^13^ while exploring a new synthesis route for ammonia borane (NH_3_BH_3_),^14–17^ we serendipitously discovered that low-temperature solid-state ammonia can be stabilized at ambient temperature. Specifically, we successfully synthesized NH_3_(s), preserved within a glass matrix, facilitated by freeze-drying an ice of boric acid and ammonium aqueous solution using liquid nitrogen at 77 K. The resulting NH_3_(s), with a lattice constant of 0.5165 nm,^13^ remained stable at ambient temperature and pressure, confirmed using X-ray diffraction (XRD), Raman spectroscopy, chemical analyses, gas and ion chromatography, and approximate thermodynamic modeling.^13^ This NH_3_(s) is a potentially safe hydrogen storage material due to its odorless nature.^13^

Investigations on ammonia stabilization contribute to the progress of planetary exploration,^18,19^ life sciences,^20^ and energy science.^2–11^ To understand the wide-ranging thermodynamic conditions in these areas, phase diagrams mapping equilibrium states as a function of temperature (*T*), pressure (*p*), and related properties have been investigated.^12,19,21–32^ In this study, the stabilization mechanism of NH_3_(s) confined in a glass matrix was investigated under ambient conditions to ensure its safety, associating it with the related phase equilibria properties.^12,19,21–32^ Specifically, the phonon densities of states (DOS) were theoretically calculated for solid-state ammonia in its ground state. Using the Debye–Einstein function based on these DOS, the standard Gibbs energy of formation (Δ_f_ *G*_m_^°^) at 298.15 K was calculated to clarify why NH_3_(s) remains stable at ambient temperature. The Δ_f_ *G*_m_^°^ data indicates that the glass matrix exerts sub-GPa-order pressure on NH_3_(s), stabilizing it in a quasi-equilibrium state. Micromechanical simulations confirmed the presence of such pressures, consistent with the thermodynamic model predictions.

## 2 Methods

### 2.1 Formation Mechanism of NH_3_(s)

Figure 1 shows the process for confining NH_3_(s) within the boric acid glass matrix, termed B_2_O_3_(gl)–B­(OH)_3_(gl), using a freeze-drying technique:^13^ (I) An aqueous solution of ammonium boric acid, prepared from commercial NH_3_(aq) and B_2_O_3_(s) at ambient temperature, is frozen at 77 K using liquid nitrogen as a refrigerant. Ammonium ions (NH_4_^+^(aq)) and metaboric acid anions (BO_2_^–^) are distributed within the ice matrix of H_2_O(s). (II) Upon vacuum evacuation, H_2_O(s) sublimates preferentially to satisfy charge neutrality, whereas NH_4_^+^(aq) condenses, failing to meet charge balance. After sufficient condensation, NH_3_(s) nucleates as the equilibrium phase at 77 K via proton transfer (H^+^(aq)) from NH_4_^+^(aq) to OH^–^(aq), forming H_2_O(s). Similarly, BO_2_^–^(aq) condenses. Subsequently, B_2_O_3_(gl) nucleates via O^2–^(aq) transfer from BO_2_^–^(aq) to neighboring H^+^(aq), also forming H_2_O(s). (III) NH_3_(s) and B_2_O_3_(gl) particles proceed to grow. (IV) A certain amount of B_2_O_3_(gl) hydrates to form B­(OH)_3_(gl), while the remaining H_2_O(s) sublimates. As a result, NH_3_(s) is confined in the boric acid glass matrix, B_2_O_3_(gl)–B­(OH)_3_(gl). Once within this characteristic structure, NH_3_(s) remains stable upon heating to ambient temperature. Chemical analysis revealed the sample contains 37 mol % NH_3_(s), 5 mol % NH_4_B_5_O_8_·4H_2_O(s), 10 mol % B_2_O_3_(gl), and 48 mol % B­(OH)_3_(gl). The average diameter of NH_3_(s) is 37.8 nm, as determined by applying the full width at half-maximum (fwhm) of the main XRD peak^13^ to the Scherrer’s equation.^33^ Based on the lattice constant of NH_3_(s) confined in the glass matrix (0.5165 nm),^13^ its density is 30 percent higher than that of liquid ammonia in pressurized vessels.

**Figure 1:**
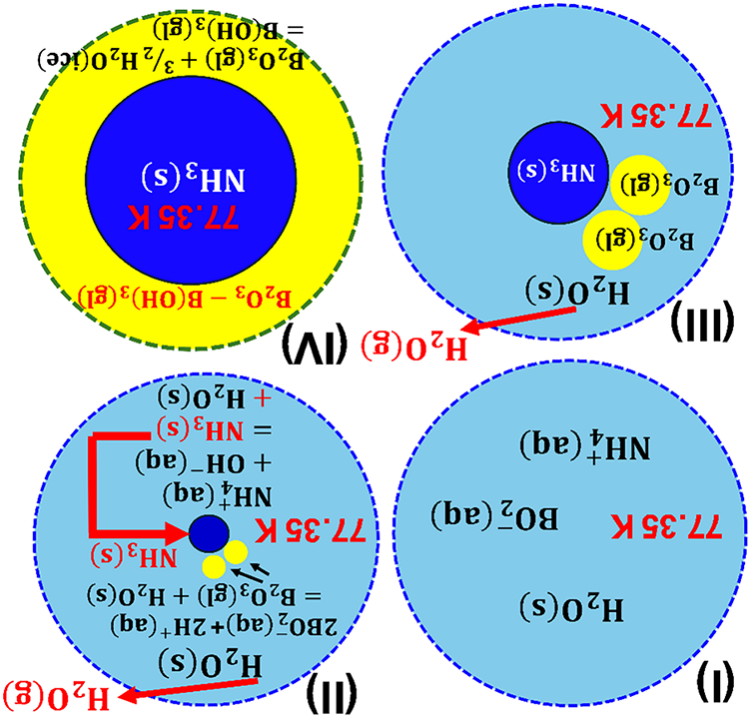
Schematic of the formation mechanism of confined NH_3_(s) at 77 K.

### 2.2 Thermodynamic Evaluation, Phonon Calculation, and Micromechanical Analysis

To determine the phase stability of NH_3_(s) at ambient temperature, its Δ_f_ *G*_m_^°^ value at 298.15 K needs to be calculated. We estimated it based on a thermodynamic cycle incorporating existing thermodynamic data as

1 $$\Delta_{f}G_{m}^{{}^{\circ}}\left({\mathrm{NH}}_{3}\left(s\right),298.15K\right)=\Delta_{f}G_{m}^{{}^{\circ}}\left({\mathrm{NH}}_{3}\left(s\right),195.4K\right)+\int_{195.4}^{298.15}C_{p,m}^{{}^{\circ}}\left({\mathrm{NH}}_{3}\left(s\right)\right)dT-\frac{1}{2}\int_{195.4}^{298.15}C_{p,m}^{{}^{\circ}}\left(N_{2}\left(g\right)\right)dT-\frac{3}{2}\int_{195.4}^{298.15}C_{p,m}^{{}^{\circ}}\left(H_{2}\left(g\right)\right)dT-T\{\int_{195.4}^{298.15}\frac{C_{p,m}^{{}^{\circ}}\left({\mathrm{NH}}_{3}\left(s\right)\right)}{T}dT-\frac{1}{2}\int_{195.4}^{298.15}\frac{C_{p,m}^{{}^{\circ}}\left(N_{2}\left(g\right)\right)}{T}dT-\frac{3}{2}\int_{195.4}^{298.15}\frac{C_{p,m}^{{}^{\circ}}\left(H_{2}\left(g\right)\right)}{T}dT\}$$

where the heat capacities of gaseous nitrogen and hydrogen, *C*_*p*,m_^°^(N_2_(g)) and *C*_*p*,m_^°^(H_2_(g)), respectively, were obtained from CODATA.^34^

At the melting point (195.4 K), the value of Δ_f_ *G*_m_^°^(NH_3_(s), 195.4 K) was determined by using *C*_*p*,m_^°^(N_2_(g)) and *C*_*p*,m_^°^(H_2_(g)), along with the heat capacities of gaseous^34^ and liquid ammonia,^35^ *C*_*p*,m_^°^(NH_3_(g)) and *C*_*p*,m_^°^(NH_3_(l)), as well as the standard Gibbs energy of formation of gaseous ammonia at 298.15 K, Δ_f_ *G*_m_^°^(NH_3_(g), 298.15 K),^34^ based on the thermodynamic cycle expressed by eqs (S1)–(S4) (see the Supporting Information (SI) for all lettered equations). Consequently, to determine Δ_f_ *G*_m_^°^(NH_3_(s), 298.15 K), the heat capacities of solid ammonia,*C*_*p*,m_^°^(NH_3_(s)), above the melting point (195.4–300 K) should be estimated.

Overstreet and Giauque^35^ previously measured *C*_*p*,m_^°^(NH_3_(s)) at 15–190 K using adiabatic calorimetry. We fitted their data in the 60–190 K range using the Debye–Einstein function^36–44^ expressed as eq 2.

2 $$C_{p,m}^{{}^{\circ}}=3\alpha R\left\{\mathrm{lD}\left(\frac{\Theta_{D}}{T}\right)+{\mathrm{mE}}_{1}\left(\frac{\Theta_{E_{1}}}{T}\right)+{\mathrm{nE}}_{2}\left(\frac{\Theta_{E_{2}}}{T}\right)\right\}+A_{1}T+A_{2}T^{3},\;71.58\;K<T<300\;K$$

The fitted function was used to estimate the *C*_*p*,m_^°^(NH_3_(s)) at 195.4–300 K, and Δ_f_ *G*_m_^°^(NH_3_(s), 298.15 K) was calculated. Here, $D\left(\frac{\Theta_{D}}{T}\right)$ is the Debye function accounting for the intermolecular translational and rotational modes of ammonia molecules in the solid crystal, NH_3_(m), whereas $E_{1}\left(\frac{\Theta_{E_{1}}}{T}\right)$ and $E_{2}\left(\frac{\Theta_{E_{2}}}{T}\right)$ are Einstein functions^45^ representing the intramolecular librational and bending modes of hydrogen atoms, H­(a). The characteristic temperatures, Θ_D_, Θ_E_1__, and Θ_E_2__, correspond to these inter- and intramolecular vibrational modes. The sum of the *T*-linear term (*A*_1_ *T*) and *T*-cubed term (*A*_2_ *T* ^3^) was used as an adjusting parameter, while the *T*-squared term (*A*_2_ *T* ^2^) in the sum is generally adopted to adjust thermal expansion.^36–44^ However, calculating *C*_*p*,m_^°^(NH_3_(s)) is complicated due to extra contributions from the orientational disorder of molecules^46–48^ and precursory phenomenon of melting.^49^ The *T*-cubed term (*A*_2_ *T* ^3^) was found to be phenomenologically preferable for including thermal expansion,^36–44^ the orientational disorder of molecules,^46–48^ and precursory phenomenon of melting^49^ described later. α represents the number of atoms per NH_3_(m) (i.e., 4).

The coefficients *l*, *m*, and *n* in eq 2, representing the relative contributions of intermolecular translational and rotational modes and intramolecular librational and bending modes, were determined from first-principles phonon calculations, which were conducted using the ALAMODE^50^ code in combination with the Vienna Ab initio Simulation Package (VASP)^51,52^ using eqs (S5)–(S8) (see the SI for computational details). For the exchange-correlation functional, the generalized gradient approximation of Perdew–Burke–Ernzerhof (GGA-PBE)^53^ was employed with the DFT-D4 dispersion correction.^54^

To complete the thermodynamic profile for NH_3_(s), low-temperature *C*_*p*,m_^°^(NH_3_(s)) data reported by Overstreet and Giauque^35^ were fitted to polynomial functions (eqs 3 and 4). To minimize fitting error, the temperature ranges were divided into three segments.^36–44^

3 $$\begin{array}{l} C_{p,m}^{{}^{\circ}}=\gamma T+\sum_{j=3,5,7,9,11}B_{i}T^{i},,0\;K<T<33.18\;K \end{array}$$

4 $$C_{p,m}^{{}^{\circ}}=\sum_{j=0}^{6}C_{j}T^{j},,33.18\;K<T<71.58\;K$$

γ and *B*_*i*_ in eq 3 are coefficients representing electronic^45,55^ and vibrational^45^ contributions to *C*_*p*,m_^○^ at very low temperatures, respectively, while *C*_*j*_ in eq 4 is an empirical coefficient for a power-law temperature function expressing *C*_*p*,m_^○^ in the intermediate temperature range. The intersection points between eqs 3 and 4, i.e., 33.18 K, and eqs 2 and 4, i.e., 71.58 K, were determined using the Newton–Raphson method.^37–43^

The likely cause of NH_3_(s) stabilization at ambient temperature is the pressure exerted by the glass matrix, resulting from a thermal expansion difference. Therefore, pressure effects were also investigated. As a fundamental thermodynamic model, the pressure required for the phase transition from gas to solid via liquid was evaluated thermodynamically. At 298.15 K, the condensation pressure was calculated using eqs (S9)–(S14) (see the SI), assuming that pressurization has a negligible effect on the chemical potential of the condensed phase.^56^ The freezing pressure was subsequently determined using eqs (S15)–(S18) (see the SI). Consequently, the total pressure required for gas-to-solid transition via the liquid state was defined as the sum (eq (S19)) of the values expressed as eqs (S14) and (S18) (see the SI).

A micromechanical simulation was conducted to confirm that the pressure predicted by this thermodynamic simulation was indeed exerted by the glass matrix on NH_3_(s). To evaluate the thermal stress acting on the solid NH_3_ particles confined within the boric glass matrix, changes in the lattice constant of NH_3_ and stress induced in the boric glass matrix surrounding the NH_3_ particles, we performed simulations based on the phase-field micromechanical elasticity theory.^57,58^ The calculations consider the constrained thermal expansion of spherical NH_3_(s) particles within the glass matrix. Here, we adopted the physical properties of pristine B_2_O_3_ since those of the glass matrix composed of 37 mol % NH_3_(s), 5 mol % NH_4_B_5_O_8_·4H_2_O(s), 10 mol % B_2_O_3_(gl), and 48 mol % B­(OH)_3_(gl) are unknown. The elastic modulus of pristine B_2_O_3_ was adopted from the experimental data of Ramos et al.^59^ For the purpose of simplification, we neglected its low thermal expansion coefficient, approximately 15 × 10^–6^ K^–1^ in the 100–300 K range.^60^ Incidentally, negative thermal expansion has been reported for B_2_O_3_ at lower temperatures.^61^ For solid NH_3_(s), the thermal expansion coefficient and elastic constant were estimated from the present first-principles calculation. The hydrostatic stress, σ_hydro_, was simulated considering theories for geometric morphologies.^62^ This analysis is further discussed in the SI, expressed as eqs (S20)–(S27) and Table S1.

## 3 Results and Discussion

Figure 2a shows the *C*_*p*,m_^○^ data for the solid (open circles) and liquid phases (open triangles) of ammonia, as measured by Overstreet and Giauque using adiabatic calorimetry.^35^ The melting of NH_3_(s) into NH_3_(l) was observed at 195.4 K, and a discontinuous increase in *C*_*p*,m_^○^ during this first-order transition was found. Moreover, the transition from NH_3_(l) to NH_3_(g) was observed at 239.7 K.^35^ The *C*_*p*,m_^○^ data for gaseous ammonia (solid squares) were evaluated from 4000 to 100 K based on statistical thermodynamic estimates from CODATA.^34^

**Figure 2:**
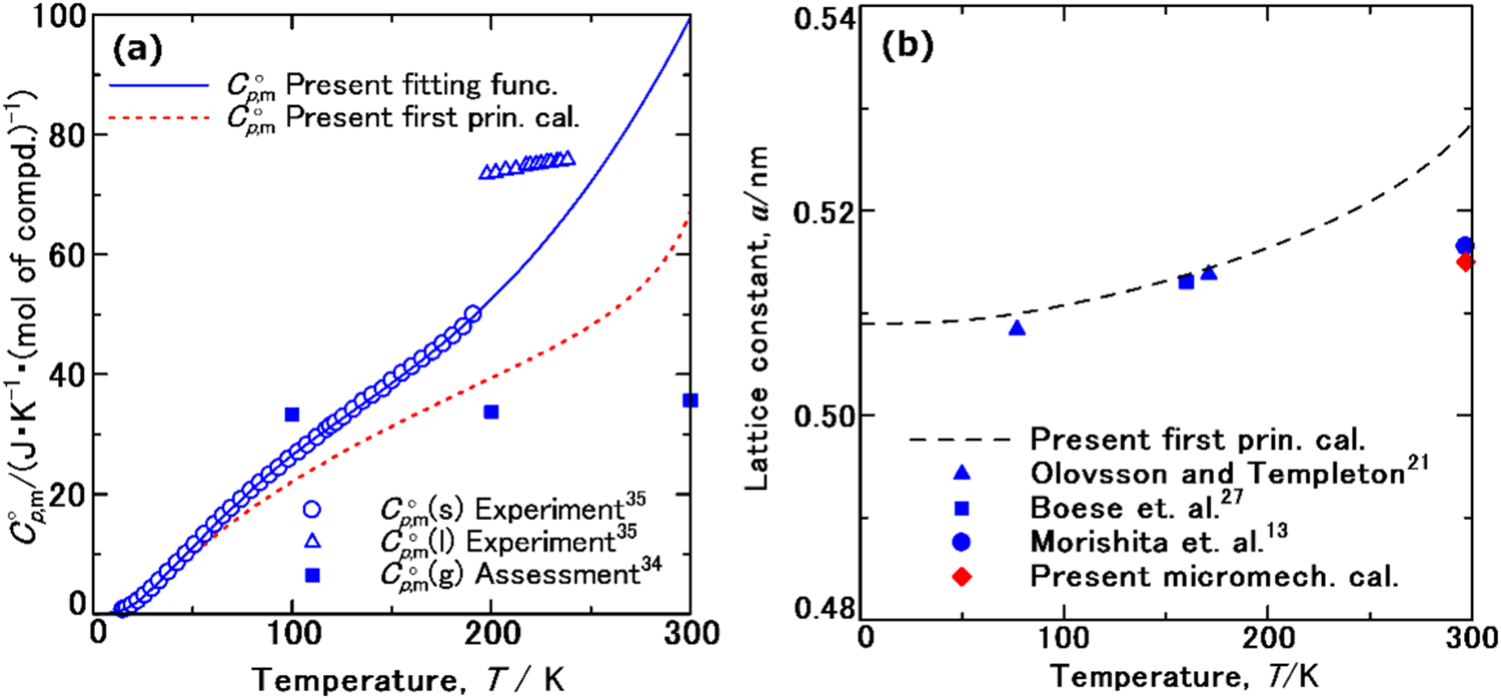
(a) *C*_*p*,m_^○^ data for ammonia: solid (open circles) and liquid (open triangles) measured by Overstreet and Giauque (Adapted in J. Am. Chem. Soc., **1937**, 59, 254–259);^35^ gas phase (solid squares) from CODATA;^34^ solid (solid line) from the Debye–Einstein function (eqs 2–4 and Table S2); solid (dotted line) simulated from our first-principles calculations. (b) Lattice constants for solid ammonia: from low-temperature in situ XRD of pristine samples by Olovson and Templeton^21^ (solid triangle) and Boese et al.^27^ (solid square); from XRD at 297.15 K using NH_3_(s) confined in a glass matrix from our previous work^13^ (solid circle); from our first-principles phonon calculation (dotted line) and micromechanical simulation (solid diamond).

The solid line in Figure 2a represents the values calculated from the fitting functions defined by eqs 2–4. Their coefficients are summarized in Table S2 (see the SI for all lettered tables). The calculated data are consistent with experimental values,^35^ and the differences between them (*u*_fit_(*C*_*p*,m_^○^)) were found to be within one percent and are shown in Figure S1 (see the SI).

The *C*_*p*,m_^○^ data for NH_3_(s) above its melting point of 195.4 K were estimated by extrapolating the Debye–Einstein function (eq 2). The slope of the extrapolated *C*_*p*,m_^○^ curve as a function of temperature increased above 195.4 K. The optimized Debye–Einstein function appeared to reflect the change in intermolecular interactions among NH_3_(m) molecules at the melting point.

Figure 2b shows the lattice constant of pristine NH_3_(s) as a function of temperature, determined from first-principles phonon calculations based on PBE + D4 ^54^ To investigate the effect of the exchange-correlation functional on the predicted lattice constants, we also conducted the calculations using PBEsol+D4,^54^ PBEsol,^63^ r^2^SCAN,^64^ and r^2^SCAN+rVV10.^65^ As shown in Figure S2 in the SI, the calculated values using PBE+D4 ^54^ were in closest agreement with experimental data.^21,27^ A similar increase in the slope of the lattice constant as a function of temperature was observed above the melting point (195.4 K). The mechanism of this increase appears to be similar to that of the *C*_*p*,m_^○^ data (Figure 2a), reflecting the changes in intermolecular interactions among NH_3_(m) molecules.

Figure 3 shows the phonon dispersion and DOS corresponding to the vibrational modes of NH_3_(s) using PBE+D4.^54^ By analyzing the computed phonon polarization vectors, the characteristic vibrational modes in each energy region were identified as follows: (I) Modes at 0–217 cm^–1^ are the intermolecular translation and rotation of NH_3_(m); (II) Modes at 313–687 cm^–1^ are the intramolecular librational vibration of H­(a); (III-A) Modes at 1096–1147 cm^–1^ are the symmetric bending vibration of H­(a); (III–B) Modes at 1611–1678 cm^–1^ are the antisymmetric bending vibration of H­(a); (IV-A) Modes at 3264–3292 cm^–1^ are the antisymmetric stretching vibration of H­(a); (IV–B) Modes at 3385–3425 cm^–1^ are the symmetric stretching vibration of H­(a).

**Figure 3:**
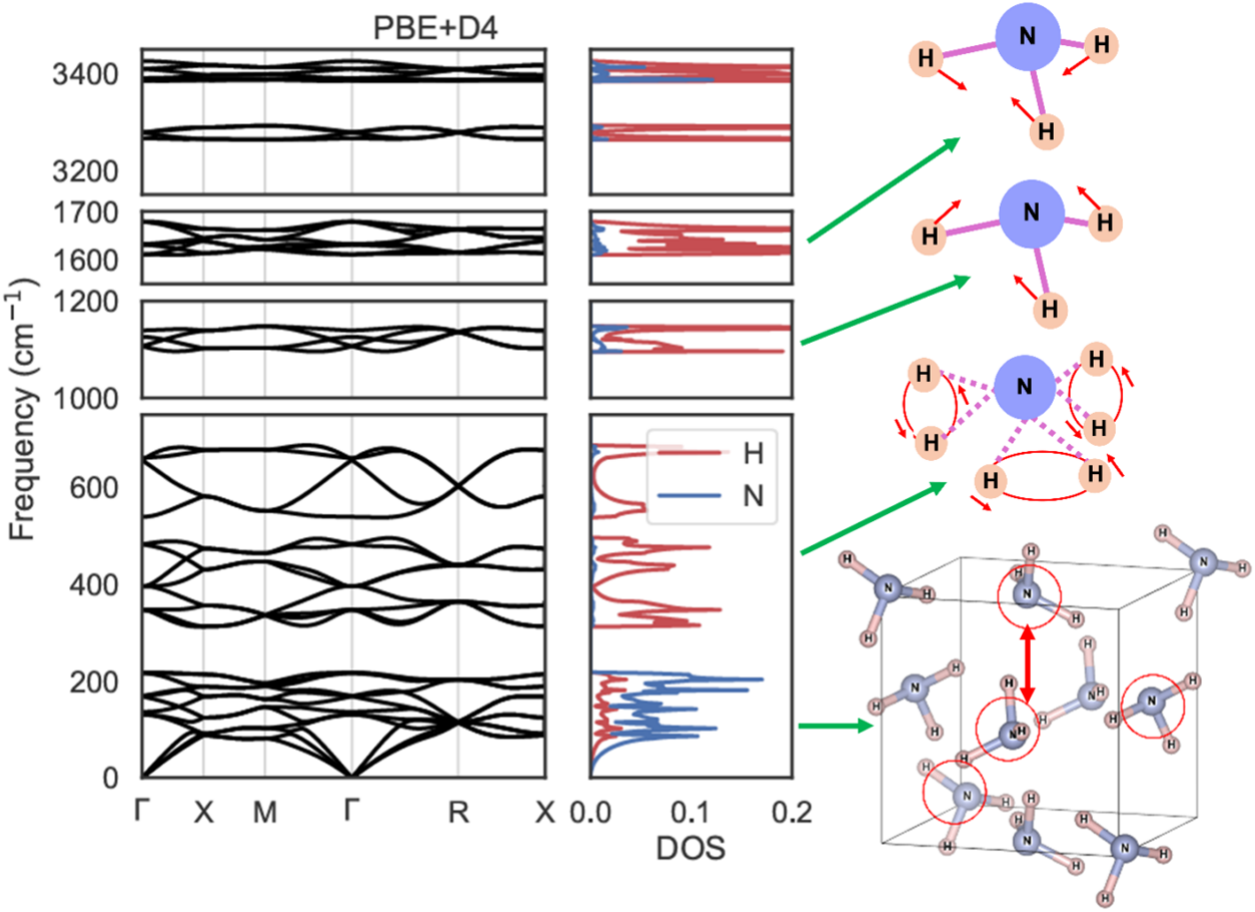
Calculated phonon dispersion and densities of states (DOS) for type I NH_3_(s). Schematic figures of vibrational modes representing each energy region are also illustrated.

To construct the Debye–Einstein function (eq 2) for extrapolating the *C*_*p*,m_^○^ data from Overstreet and Giauque,^35^ the relative ratios *l*, *m*, and *n* were determined based on the vibrational modes described above: (a) Because $D\left(\frac{\Theta_{D}}{T}\right)$ is defined as the acoustic mode due to coupled atomic motion,^45^ it is appropriate to evaluate the intermolecular translational and rotational modes of NH_3_(m) at a lower wavenumber range (0–217 cm^–1^). The relative ratio of the sum of the partial densities of states (PDOS) of N­(a) and H­(a) at 0–217 cm^–1^ to the full DOS contributing to the *C*_*p*,m_^○^ was 0.33270. Thus, *l*, representing the relative ratio of $D\left(\frac{\Theta_{D}}{T}\right)$ , was assigned the value of 0.33270. (b) Optical modes in the 313–687 cm^–1^ range, associated with the librational vibration of H­(a), were evaluated using $E_{1}\left(\frac{\Theta_{E_{1}}}{T}\right)$ . The relative PDOS ratio was 0.33378, which was used for *m*, representing the relative ratio of $E_{1}\left(\frac{\Theta_{E_{1}}}{T}\right)$ . (c) $E_{2}\left(\frac{\Theta_{E_{2}}}{T}\right)$ was used to evaluate the sum of the optical modes at 1096–1147 and 1611–1678 cm^–1^, corresponding to the symmetric and antisymmetric vibrations of H­(a). The relative PDOS ratio was 0.33349, and this was adopted for *n*, representing the relative ratio of $E_{2}\left(\frac{\Theta_{E_{2}}}{T}\right)$ . The wavenumber ranges 3264–3292 and 3385–3425 cm^–1^, corresponding to antisymmetric and symmetric stretching vibrations, exceed the detection limits of *C*_*p*,m_^○^ measurements; therefore, these were excluded from the model. As a result, the Debye–Einstein function incorporating these *l*, *m*, and *n* values showed good agreement with the experimental data of Overstreet and Giauque.^35^

The *C*_*p*,m_^○^ data determined from first-principles calculations are depicted with a dotted line in Figure 2a and solid lines in Figure S3. Popov et al.^48^ measured the *C*_*p*,m_^○^ data for NH_3_(s) and confirmed the accuracy of the data of Overstreet and Giauque^35^ noting that extra heat capacity was generated due to the orientational disorder of NH_3_(s) molecules. Additional contributions to the heat capacity are also known to occur due to the precursory phenomena of melting.^49^ The theoretical values were found to be smaller than the experimental data.^35^ We confirmed that the calculated *C*_*p*,m_^○^ values are consistently smaller than the experimental ones for all the considered exchange-correlation functionals. Hence, the underestimation is potentially due to contributions not only from the orientational disorder of NH_3_ molecules^46–48^ or anharmonic effects but also from the precursory phenomenon of melting,^49^ which is not captured in the present phonon calculations.

Figure 4a shows the Δ_f_ *G*_m_^°^ data at 100–300 K for solid, liquid, and gaseous ammonia. Above the boiling point, the Δ_f_ *G*_m_^°^ values were more negative in the order of gas, liquid, and solid, consistent with the hierarchy of their phase stability. However, the Δ_f_ *G*_m_^°^ data at ambient temperatures for solid ammonia were found to be negative, indicating that it can exist in a quasi-equilibrium state. Thermodynamic data for solid, liquid, and gas phases were summarized in Tables S3 and S4 (see the SI). At 298.15 K, the Δ_f_ *G*_m_^°^ data for solid, liquid, and gaseous ammonia were calculated as

5 $$\Delta_{f}G_{m}^{{}^{\circ}}\left({\mathrm{NH}}_{3}\left(s\right),298.15\;K\right)/\left({\mathrm{kJ}\left(\mathrm{mol of compd}.\right)}^{-1}\right)=-7.650$$

6 $$\Delta_{f}G_{m}^{{}^{\circ}}\left({\mathrm{NH}}_{3}\left(l\right),298.15\;K\right)/\left({\mathrm{kJ}\left(\mathrm{mol of compd}.\right)}^{-1}\right)=-11.104$$

7 $$\Delta_{f}G_{m}^{{}^{\circ}}\left({\mathrm{NH}}_{3}\left(g\right),298.15\;K\right)/\left({\mathrm{kJ}\left(\mathrm{mol of compd}.\right)}^{-1}\right)=-16.319$$

**Figure 4:**
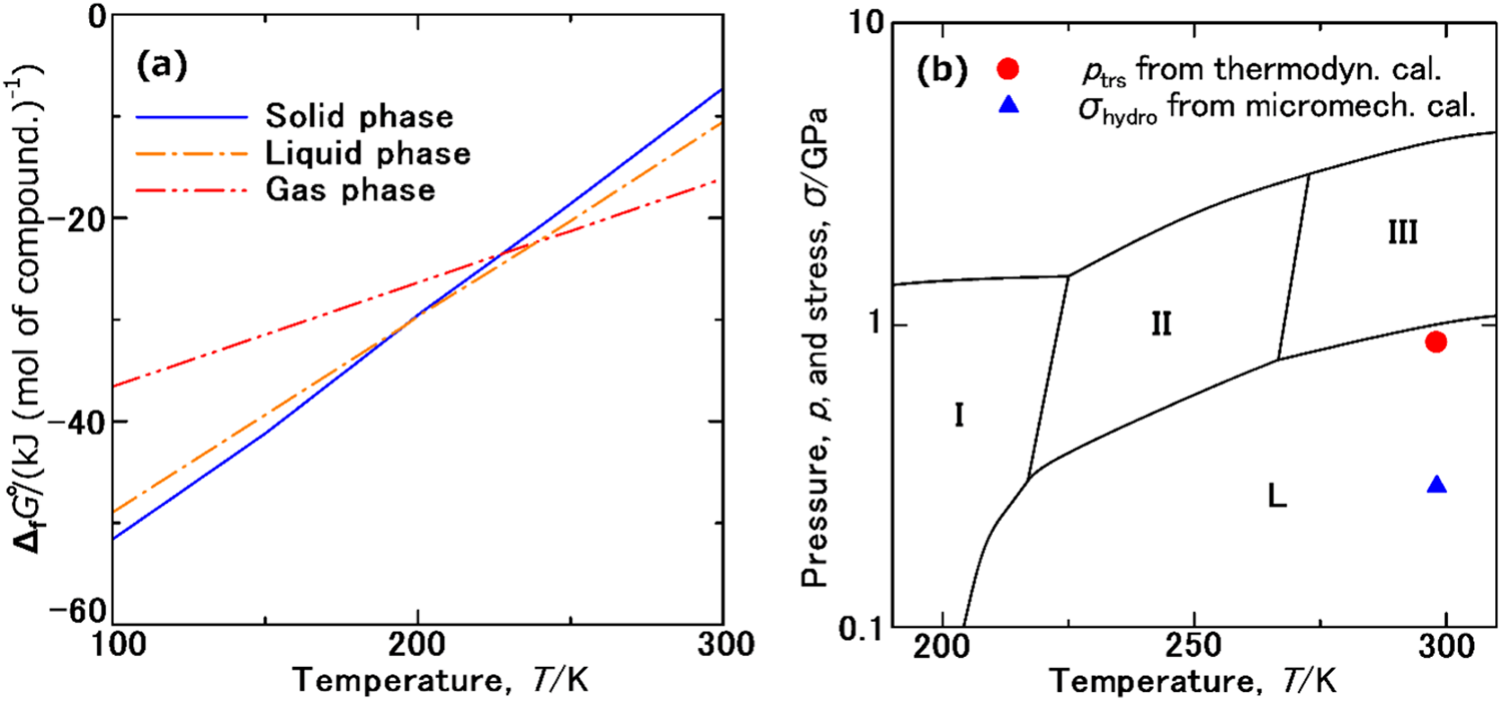
(a) Δ_f_ *G*_m_^°^ data at 100–300 K for solid (solid line), liquid (dashed-dotted line), and gaseous ammonia (dashed double-dotted line). (b) Pressure, *p*_trs_, and hydrostatic stress, σ_hydro_, required for the phase transition based on our thermodynamic calculations (solid circle) and micromechanical simulation (solid triangle) on the *p–T* phase diagram of ammonia.^26^ (Adapted in part in Phys. Rev. Lett., **1995**, 76, 74–77).

From these Δ_f_ *G*_m_^°^ values, the Gibbs energies of condensation, Δ_con_ *G*_m_^°^, freezing, Δ_freez_ *G*_m_^°^, and phase transition from the gas to solid phase via the liquid phase, Δ_trs_ *G*_m_^°^, at 298.15 K were estimated as

8 $$\Delta_{\mathrm{con}}G_{m}^{{}^{\circ}}\left({\mathrm{NH}}_{3},298.15\;K\right)/\left({\mathrm{kJ}\left(\mathrm{mol of compd}.\right)}^{-1}\right)=5.414$$

9 $$\Delta_{\mathrm{freez}}G_{m}^{{}^{\circ}}\left({\mathrm{NH}}_{3},298.15\;K\right)/\left({\mathrm{kJ}\left(\mathrm{mol of compd}.\right)}^{-1}\right)=3.255$$

10 $$\Delta_{\mathrm{trs}}G_{m}^{{}^{\circ}}\left({\mathrm{NH}}_{3},298.15\;K\right)/\left({\mathrm{kJ}\left(\mathrm{mol of compd}.\right)}^{-1}\right)=8.669$$

As given by eqs (S14), (S18), and (S19) in the SI, the pressures required for condensation, *p*_con_, freezing, *p*_con_, and phase transition from the gas to solid phase via the liquid phase, *p*_trs_, were estimated as

11 $$p_{\mathrm{con}}\left({\mathrm{NH}}_{3},298.15\;K\right)/\mathrm{GPa}=8.88\times 10^{-4}$$

12 $$p_{\mathrm{freez}}\left({\mathrm{NH}}_{3},298.15\;K\right)/\mathrm{GPa}=0.874$$

13 $$p_{\mathrm{trs}}\left({\mathrm{NH}}_{3},298.15\;K\right)/\mathrm{GPa}=0.875$$

Figure 4b shows the phase diagram of ammonia under high pressure.^26^ Complex polymorphs from phase I to phase III were observed in the solid state. Phase I was found to be a cubic crystal (*P*2_1_3),^21,25–27^ corresponding to the NH_3_(s) confined in the glass matrix in our previous study.^13^ Phase II was identified as a hexagonal crystal (*P*6_3_/*mmc*),^25^ while phase III was cubic (*Fm*3*m*),^23,25,26^ representing a structure where the reciprocal rotation of NH_3_(m) in phase I decreases the degree of order. When we plotted the *p*_trs_ value determined from the gas to solid transition via the liquid phase (solid circle in Figure 4b), it was well correlated with the phase boundary between the liquid phase and solid phase III. However, as mentioned above, the NH_3_(s) from our previous study^13^ was determined to be cubic phase I (*P*2_1_3),^21,25–27^ and not cubic phase III (*Fm*3*m*).^23,25,26^ Various polymorphs of fine particles are generally known to be formed under ambient conditions. Structures of α- and γ- type polymorphs in alumina (Al_2_O_3_)^66^ and rutile- and anatase-type polymorphs in titania (TiO_2_)^67^ are formed depending on particle size, surface energy and the surface adsorption of moisture. Based on these polymorphs, the nanocrystal of ammonia cubic phase I^13^ is likely to be stable within the glass matrix. Robust B–N bonds are likely to form along the interface between the cubic phase I and the glass matrix. The interfacial energy originating from a B–N bonds is likely to contribute to the configuration of the cubic phase I.

To clarify that *p*_trs_ acts on solid NH_3_ particles, we performed a simulation based on the phase-field micromechanical elasticity theory.^57,58^ While lattice parameters obtained from PBE+D4 calculations accurately reproduced previously reported values at low temperatures,^21,27^ discrepancies were observed when compared to the lattice constant of solid ammonia in the glass matrix measured at 297 K.^13^ This is likely due to the thermal expansion of solid NH_3_ particles being elastically constrained by the glass matrix. Hence, the lattice parameter of solid NH_3_ particles within the glass matrix, hydrostatic stress, σ_hydro_, experienced by solid NH_3_ particles, and maximum principal stress, σ_max_, acting on the glass matrix were calculated using eqs (S20)–(S27) using the model illustrated in Figure S4 (see the SI). The simulation assumed a zero-strain state for the solid NH_3_ particles within the glass matrix at 77 K and calculated the stress generated upon heating to 297 K. The result, indicated by the red diamond in Figure 2b, corresponds to a lattice parameter of 0.5150 nm, which is closer to the experimentally determined value of 0.5165 nm.^13^ The estimated σ_hydro_ and σ_max_ are summarized in Table S5 (see the SI). Plotting σ_hydro_ (solid triangle) in Figure 4b indicates that the NH_3_ particles experience compressive sub-GPa stress within the glass matrix consistent with the order of *p*_trs_ predicted from the thermodynamic estimation. Additional pressure likely results from interfacial tension between the solid NH_3_ particles and boric glass matrix, as defined by the Young–Laplace equation.^68^ Although the interfacial tension between solid NH_3_(s) and the glass matrix is unknown, considering the nanoparticle size of NH_3_(s) (37.8 nm), a certain additional hydrostatic stress can be expected.

Tschauner et al.^69^ clarified that ice-VII is confined in natural diamond as inclusions and serves as an indicator for water-rich regions in Earth’s deeper mantle. Ice-VII is a high-pressure form of water ice that is stable above 2.4 GPa.^69^ This means robust matrices can confine substances which are stable under high pressures. The theoretical strength of a perfect crystal is defined as the force required to induce failure beyond interatomic interactions in an ideal crystal^70^ and is estimated to be ∼ 9 GPa based on theoretical analysis of interatomic potentials. In contrast, the practical strengths of glasses are often reduced to one-hundredth or 1000th of their theoretical values due to the inherent formation of micro cracks that lead to failure.^71,72^ The estimated σ_max_ value of 0.122 GPa appears to exceed the typical strengths of practical glasses.^71,72^ However, localized nanosized regions in the glass matrix surrounding the NH_3_ nanoparticles appear to exhibit a microcrack-free structure. As a result, the glass matrix likely endures the local σ_max_ caused by the difference in thermal expansion coefficient between itself and the NH_3_ particles. Thus, one of the key reasons for the stabilization of NH_3_(s) at ambient temperature is its confinement within an amorphous glass structure.

Beyond this pressure effect, the role of phase equilibria governing NH_3_(s), the glass matrix (GM­(gl)), and the impurity phase NH_4_B_5_O_8_·4H_2_O(s) warrants further investigation. In the pseudo ternary NH_3_(s)–GM­(gl)–NH_4_B_5_O_8_·4H_2_O(s) system, the solidus surface is generally known to shift to higher temperatures when a peritectic reaction occurs. To quantitatively evaluate this behavior, it is essential to determine not only the values of Δ_mix_ *G*_m_^°^ for GM­(gl) and Δ_f_ *G*_m_^°^ for NH_4_B_5_O_8_·4H_2_O(s) but also the Gibbs energy of substitution of boron into the nitrogen sites in NH_3_(s). These unknown thermodynamic parameters must be systematically estimated in future studies to enable accurate phase equilibrium modeling and deepen our understanding of the N*H*_3_(*s*) stabilization mechanism.

## 4 Conclusion

The current study elucidated the stabilization mechanism of solid-state ammonia, NH_3_(s), confined within a boric acid glass matrix using a thermodynamic model supported by first-principles phonon calculation and hydrostatic stress analysis by micromechanics. Phonon calculations revealed vibrational DOS for the intermolecular translational and rotational modes of NH_3_ molecules and intramolecular librational and bending modes of hydrogen atoms. The heat capacity of NH_3_(s), *C*_*p*,m_^○^, was calculated from a Debye function accounting for the intermolecular translational and rotational modes of NH_3_ and two Einstein functions accounting for the intramolecular librational and bending modes of hydrogen, regarding the DOS for these modes as the contribution ratios of the three functions. The standard Gibbs energy of formation of NH_3_(s), Δ_f_ *G*_m_^°^, at 298.15 K, determined by inserting the optimum *C*_*p*,m_^○^ function into the thermodynamic cycle, was −7.650 (kJ­(mol of compd.)^−1^). From this value, the standard Gibbs energy of transition from gas to solid via liquid, Δ_trs_ *G*_m_^°^, was calculated to be 8.669 (kJ­(mol of compd.)^−1^). Consequently, the pressure required for this phase transition, *p*_trs_, was estimated to be in the order of sub-GPa. Micromechanical analysis indicated that this hydrostatic stress originates from the difference between the thermal expansion coefficients of NH_3_(s) and the glass matrix and stabilizes the ammonia crystals within the matrix. The present findings open a new avenue for stabilizing nonequilibrium phases at ambient temperature. A future challenge for the practical application of solid ammonia is the discovery of an outer matrix that can facilitate the evolution of H_2_(g) from the decomposition of NH_3_(s) confined within the glass matrix.

## Supplementary Material



## Data Availability

All data generated or analyzed during this study are included in the published article and its Supporting Information.

## Notes

Detailed methods and results for thermodynamic calculation, phonon mode simulation, and micromechanical pressure analysis (PDF)
