# Supplementary material for: Mechanism of Solid Ammonia Stabilization at Ambient Temperature: Insights from Thermodynamics, Phonon Calculation, and Micromechanics
Source: ACS Omega. 2025 Nov 17;10(47):57684–91. doi: 10.1021/acsomega.5c09165 (PMC12703987; doi:10.1021/acsomega.5c09165)
Supplement: Supplementary file 1 [file ao5c09165_si_001.pdf]

Mechanism of Solid Ammonia Stabilization at Ambient Temperature: Insights from Thermodynamics, Phonon Calculation, and Micromechanics

Masao Morishita [a], Terumasa Tadano [b], Yusuke. Matsuoka [a], and Taichi Abe [a]*

[a] Research Center for Structural Materials, National Institute for Materials Science, 1-2-1 Sengen, Tsukuba, Ibaraki 305-0047, Japan

[b] Research Center for Magnetic and Spintronic Materials, National Institute for Materials Science, 1-2-1 Sengen, Tsukuba, Ibaraki 305-0047, Japan

*Email: MORISHITA.Masao@nims.go.jp

Supporting Information

METHODS

Assessment of thermodynamic data for solid-state ammonia

At the boiling point (239.7 K), the standard Gibbs energy of formation, $\Delta_f G_m^\circ$, of gaseous ammonia, $NH_3(g)$, is represented by Equation (S1):

$$\Delta_f G_m^\circ(NH_3(g),\ 239.7\ K) = \Delta_f G_m^\circ(NH_3(g),\ 298.15\ K) + \int_{298.15}^{239.7} C_{p,m}^\circ(NH_3(g))dT$$
$$-\frac{1}{2}\int_{298.15}^{239.7} C_{p,m}^\circ(N_2(g))dT - \frac{3}{2}\int_{298.15}^{239.7} C_{p,m}^\circ(H_2(g))dT \qquad (S1)$$
$$-T\left\{\int_{298.15}^{239.7}\frac{C_{p,m}^\circ(NH_3(g))}{T}dT - \frac{1}{2}\int_{298.15}^{239.7}\frac{C_{p,m}^\circ(N_2(g))}{T}dT - \frac{3}{2}\int_{298.15}^{239.7}\frac{C_{p,m}^\circ(H_2(g))}{T}dT\right\}.$$

Liquid ammonia, $NH_3(l)$, equilibrates with $NH_3(g)$ at the boiling point. Therefore, the $\Delta_f G_m^\circ$ of the liquid phase is equal to that of the gas phase, as seen in Equation (S2):

$$\Delta_f G_m^\circ(NH_3(l),\ 239.7\ K) = \Delta_f G_m^\circ(NH_3(g),\ 239.7\ K). \qquad (S2)$$

At the melting point (195.4 K), the $\Delta_f G_m^\circ$ of the liquid phase is represented by Equation (S3):

$$\Delta_f G_m^\circ(NH_3(l),\ 195.4\ K) = \Delta_f G_m^\circ(NH_3(l),\ 239.7\ K) + \int_{239.7}^{195.4} C_{p,m}^\circ(NH_3(l))dT$$
$$-\frac{1}{2}\int_{239.7}^{195.4} C_{p,m}^\circ(N_2(g))dT - \frac{3}{2}\int_{239.7}^{195.4} C_{p,m}^\circ(H_2(g))dT$$
$$-T\left\{\int_{239.7}^{195.4}\frac{C_{p,m}^\circ(NH_3(l))}{T}dT - \frac{1}{2}\int_{239.7}^{195.4}\frac{C_{p,m}^\circ(N_2(g))}{T}dT - \frac{3}{2}\int_{239.7}^{195.4}\frac{C_{p,m}^\circ(H_2(g))}{T}dT\right\}. \qquad (S3)$$

Solid-phase ammonia, $NH_3(s)$, equilibrates with $NH_3(l)$ at the boiling point. Therefore, the $\Delta_f G_m^\circ$ of the solid phase is equal to that of the liquid phase, as seen in Equation (S4),

$$\Delta_f G_m^\circ(NH_3(s),\ 195.4\ K) = \Delta_f G_m^\circ(NH_3(l),\ 195.4\ K). \tag{S4}$$

In Equations (S1)–(S4), values for $\Delta_f G_m^\circ(NH_3(g),\ 298.15\ K), C_{p,m}^\circ(NH_3(g))$, $C_{p,m}^\circ(N_2(g))$, and $C_{p,m}^\circ(H_2(g))$ were adopted from CODATA,[34] while the value for $\Delta_f G_m^\circ(NH_3(l))$ was taken from the report by Overstreet and Giauque.[35]

Computational details of first-principles calculation

To compute the phonon band structure, density of states (DOS), and the temperature-dependent lattice constants of the type-I solid NH_3, we conducted first-principles calculations based on density functional theory (DFT) using the *Vienna Ab initio Simulation Package* (VASP)[51,52]. First, we optimized the lattice parameters and internal atomic coordinates until the residual forces on all atoms were below 0.001 eV/Å. We employed a kinetic energy cutoff of 520 eV, a convergence tolerance of 10^{-8} eV for self-consistent field iterations, and a $9 \times 9 \times 9$ Gamma-centered k points for the Brillouin zone sampling. Subsequently, we constructed a $3 \times 3 \times 3$ supercell containing 432 atoms from the optimized primitive cell. Each atom in this supercell was displaced from its equilibrium position by 0.01 Å, and the resulting atomic forces were calculated using VASP. The second-order interatomic force constants (IFCs) were determined by fitting the displacement-force dataset. Phonon frequencies $\{\omega_{\boldsymbol{q}\nu}\}$ within the harmonic approximation were obtained by diagonalizing the dynamical matrix constructed from these IFCs. To account for the long-range dipole-dipole interaction, we employed the Ewald summation technique using the dielectric tensor and the Born effective charges computed using density functional perturbation theory (DFPT). The IFC and phonon calculations were performed using the ALAMODE software[50].

To obtain the temperature-dependent lattice constants $a(T)$, bulk modulus $B(T)$, and the heat capacity at constant pressure $C_p(T)$, we performed phonon calculations at 11 different lattice constants: $a_0, 1.01a_0, 1.02a_0, \dots, 1.10a_0$, where a_0 is the DFT-optimized lattice constant. From

the calculated electronic ground-state energy $U_0(a_i)$ and phonon frequencies $\{\omega_{\boldsymbol{q}\nu}(a_i)\}$, the Helmholtz free energy at lattice constant a_i (the corresponding volume V_i) was obtained as

$$F(V_i,T) = U_0(V_i) + F_{\mathrm{vib}}(V_i,T), \tag{S5}$$

where $F_{\mathrm{vib}}(V,T)$ is the vibrational free energy defined as

$$F_{\mathrm{vib}}(V,T) = \sum_{\boldsymbol{q}\nu}\left[\frac{\hbar\omega_{\boldsymbol{q}\nu}(V)}{2} + k_{\mathrm{B}}T\ln\left(1 - e^{-\hbar\omega_{\boldsymbol{q}\nu}(V)/k_{\mathrm{B}}T}\right)\right]. \tag{S6}$$

A q point mesh of $30 \times 30 \times 30$ was employed in Eq. (S6). The equilibrium volume (V_{eq}), bulk modulus (B_{eq}), and its pressure derivative ($B_{eq}^{'}$) were determined by fitting the calculated free energies $F(V_i,T)$ to the Birch–Murnaghan equation of state:

$$E(\eta) = E_0 + \frac{9B_{\mathrm{eq}}V_{\mathrm{eq}}}{16}(\eta^2-1)^2\left[6 + B_{\mathrm{eq}}'(\eta^2-1) - 4\eta^2\right]. \tag{S7}$$

Here, $\eta = (V/V_0)^{1/3} = a/a_0$. By repeating the above procedure across various temperatures, we obtained the temperature-dependent lattice constants $a(T)$ and bulk modulus $B(T)$. The heat capacity $C_p(T)$ was evaluated as

$$C_p(T,p) = C_V(V(p,T),T) + T\frac{\partial V(p,T)}{\partial T}\frac{\partial S(V,T)}{\partial V}|_{V=V(p,T)}, \tag{S8}$$

where C_V and $\partial S/\partial V$ at $V = V(p,T)$ were obtained by fitting polynomial functions to discrete values of $C_V(V_i)$ and vibrational entropy $S_{\mathrm{vib}}(V_i)$.

Assessment of pressure to stabilize solid-state ammonia at ambient temperature

At 298.15 K, the change in Gibbs energy of condensation under the pressure p_{con}, which drives the transition from gas to liquid, is expressed as Equation (S9):

$$\Delta G_p(\mathrm{g} \rightarrow \mathrm{l}) = \{\mu_\mathrm{f}(\mathrm{l}) - \mu^\circ(\mathrm{l})\} - \{\mu_\mathrm{f}(\mathrm{g}) - \mu^\circ(\mathrm{g})\} = -RT\ln\frac{p_{\mathrm{con}}}{p^\circ}, \qquad \text{(S9)}$$

where the first term, $\{\mu_\mathrm{f}(\mathrm{l}) - \mu^\circ(\mathrm{l})\}$, is the change in the chemical potential of liquid-phase due to pressurization. Generally, the change in the chemical potential of a condensed phase under pressing during condensation is negligibly small.[56] Therefore, this first term was neglected. The second term, $\{\mu_\mathrm{f}(\mathrm{g}) - \mu^\circ(\mathrm{g})\}$, denotes the change in the chemical potential of the gas phase with pressurization and is expressed as $RT\ln\frac{p_\mathrm{f}}{p^\circ}$. On the other hand, at 298.15 K, the standard Gibbs energy of condensation, $\Delta_{\mathrm{con}}G_\mathrm{m}^\circ$, from liquid to solid is defined by Equation (S10):

$$\Delta_{\mathrm{con}}G_\mathrm{m}^\circ = \Delta_\mathrm{f}G_\mathrm{m}^\circ(\mathrm{NH_3(l)},\ 298.15\ \mathrm{K}) - \Delta_\mathrm{f}G_\mathrm{m}^\circ(\mathrm{NH_3(g)},\ 298.15\ \mathrm{K}). \qquad \text{(S10)}$$

The first term, $\Delta_\mathrm{f}G_\mathrm{m}^\circ(\mathrm{NH_3(l)},\ 298.15\ \mathrm{K})$, is defined by Equation (S11):

$$\Delta_\mathrm{f}G_\mathrm{m}^\circ(\mathrm{NH_3(l)},\ 298.15\ \mathrm{K}) = \Delta_\mathrm{f}G_\mathrm{m}^\circ(\mathrm{NH_3(l)},\ 195.4\ \mathrm{K}) + \int_{195.4}^{298.15} C_{p,\mathrm{m}}^\circ\big(\mathrm{NH_3(l)}\big)\mathrm{d}T$$

$$-\frac{1}{2}\int_{195.4}^{298.15} C_{p,\mathrm{m}}^\circ\big(\mathrm{N_2(g)}\big)\mathrm{d}T - \frac{3}{2}\int_{195.4}^{298.15} C_{p,\mathrm{m}}^\circ\big(\mathrm{H_2(g)}\big)\mathrm{d}T \qquad \text{(S11)}$$

$$-T\left\{\int_{195.4}^{298.15} \frac{C_{p,\mathrm{m}}^\circ\big(\mathrm{NH_3(l)}\big)}{T}\mathrm{d}T - \frac{1}{2}\int_{195.4}^{298.15} \frac{C_{p,\mathrm{m}}^\circ\big(\mathrm{N_2(g)}\big)}{T}\mathrm{d}T - \frac{3}{2}\int_{195.4}^{298.15} \frac{C_{p,\mathrm{m}}^\circ\big(\mathrm{H_2(g)}\big)}{T}\mathrm{d}T\right\}.$$

Here, the first term of right side, $\Delta_f G_m^\circ(NH_3(l),\ 195.4\ K)$, is given by Equation (S11) described above. The extrapolated function fitting $C_{p,m}^\circ(NH_3(l))$[35] was optimized and expressed in the footnote of Table S2 below.

Thus, $\Delta_f G_m^\circ(NH_3(l),\ 298.15\ K)$ was determined by inserting $\Delta_f G_m^\circ(NH_3(l),\ 195.4\ K)$ and the optimized fitting function of $C_{p,m}^\circ(NH_3(l))$ into Equation (S11). Hence, $\Delta_{con} G_m^\circ$ was determined.

Equating $\Delta_{con} G_m^\circ$ with $\Delta G_p(g \to l)$ is expressed in Equation (S12):

$$\Delta_{con} G_m^\circ + \Delta G_p(g \to l) = 0. \tag{S12}$$

Consequently, $\Delta_{con} G_m^\circ$ can be written as Equation (S13):

$$\Delta_{con} G_m^\circ = \{\mu_f(g) - \mu^\circ(g)\} = RT \ln \frac{p_{con}}{p^\circ}. \tag{S13}$$

From here, Equation (C5) is rewritten as Equation (S14):

$$\frac{p_{con}}{p^\circ} = \exp\left(\frac{\Delta_{con} G_m^\circ}{RT}\right). \tag{S14}$$

Then, at 298.15 K, the change in Gibbs energy of freezing under the pressure p_{freez}, driving the transition from liquid to solid, is expressed as Equation (S15):

$$\begin{aligned} \Delta G_p(l \to s) &= \{\mu_f(s) - \mu^\circ(s)\} - \{\mu_f(l) - \mu^\circ(l)\} \\ &= V_m(s) \int_{p^\circ}^{p_{freez}} dp - V_m(l) \int_{p^\circ}^{p_{freez}} dp \qquad \text{(S15)} \\ &= \{V_m(s) - V_m(l)\} \times \int_{p^\circ}^{p_{freez}} dp \\ &= \{V_m(s) - V_m(l)\} \times (p_{freez} - p^\circ) \end{aligned}$$

where $V_m(s)$ and $V_m(l)$ are the molar volumes of the solid and liquid phases, respectively. $V_m(s)$ was taken as 2.07444×10^{-5} m^3 based on the described $NH_3(s)$ confined in the glass matrix (GM) with a lattice constant of 0.5165 nm in our previous study.[13] $V_m(l)$ was taken as 2.44690×10^{-5} m^3 from the reference data.[13] Therefore, the volume reduced by 3.72457×10^{6} m^3 through freezing. Although the $V_m(s)$ and $V_m(l)$ values were measured at 297.15 and 293.15 K, respectively, they were approximated as values at 298.15 K for our thermodynamic analyses.

The standard Gibbs energy of freezing, $\Delta_{freez}G_m^\circ$, is represented by Equation (S16):

$$\Delta_{freez}G_m^\circ = \Delta_f G_m^\circ(NH_3(s),\ 298.15\ K) - \Delta_f G_m^\circ(NH_3(l),\ 298.15\ K). \tag{S16}$$

Equating $\Delta_{freez}G_m^\circ$ with $\Delta G_p(l \rightarrow s)$ provides Equation (S17):

$$\Delta_{freez}G_m^\circ + \Delta G_p(l \rightarrow s) = 0. \tag{S17}$$

Consequently, from Equations (S15)–(S17), the pressure, p_{freez}, driving the liquid to solid transition is given by Equation (S18):

$$p_{freez} = \frac{\Delta_{freez}G_m^\circ}{\{V_m(cr) - V_m(l)\}} + p^\circ. \tag{S18}$$

Hence, the pressure, p_{trs}, required to form solid from gas via the liquid phase is given by Equation (S19):

$$p_{sol} = p_{con} + p_{freez}. \tag{S19}$$

Detail of micromechanical calculation

The stress field is described by the Hooke's law:

$$\sigma_{ij}(r) = C_{ijkl}(r)\varepsilon_{kl}^{e}(r) \quad \text{(S20)}$$

where σ_{ij} represents the stress tensor, C_{ijkl} is the local elastic modulus tensor, and ε_{kl}^{e} is the local elastic strain tensor.

The elastic modulus is expressed as

$$C_{ijkl}(r) = C_{ijkl}^{0} + \Delta C_{ijkl}\phi(r) \quad \text{(S21)}$$

Here, C_{ijkl}^{0} represents the elastic modulus of the B_2O_3 ($C_{ijkl}^{B_2O_3}$), and ΔC_{ijkl} denotes the difference in elastic moduli between B_2O_3 and NH_3 ($\Delta C_{ijkl} = C_{ijkl}^{B_2O_3} - C_{ijkl}^{NH_3}$). The function ϕ is a spatial indicator, with $\phi = 1$ inside the spherical particles and $\phi = 0$ outside. The elastic strain ε_{ij}^{e} is defined as

$$\varepsilon_{ij}^{e}(r) = \bar{\varepsilon}_{ij} + e_{ij}^{c}(r) - \varepsilon_{ij}^{0}(r) \quad \text{(S22)}$$

where $\bar{\varepsilon}_{ij}$ represents a uniform strain representing the average strain across the simulation domain, e_{ij}^{c} is the deviation from the average, computed using an iterative perturbation method,[58] and ε_{ij}^{0} is the eigenstrain. The thermal expansion of NH_3 is incorporated through the eigenstrain term:

$$\varepsilon_{ij}^{0}(r) = \eta\delta_{ij}\phi(r) \quad \text{(S23)}$$

where η represents the volumetric expansion of NH_3 due to temperature increase from 77 K to 297 K, and δ_{ij} is the Kronecker delta. η is calculated as

$$\eta = \frac{a_{297\text{K}} - a_{77\text{K}}}{a_{77\text{K}}} \tag{S24}$$

The pressure within the particles was determined from the calculated stress field σ_{ij} as the hydrostatic stress, σ_{hydro}, at the particle center:

$$\sigma_{\text{hydro}} = \frac{\sigma_{11} + \sigma_{22} + \sigma_{33}}{3} \tag{S25}$$

The change in the NH_3 lattice constant due to elastic strain was calculated using the elastic strain at the particle center $\varepsilon_{ij}^{\text{e}}$:

$$a_{297\text{K}}^{\text{strained}} = a_{77\text{K}}(1 + \varepsilon_{11}^{\text{e}} + \varepsilon^{0}) \tag{S26}$$

Note that in the current configuration of isotropic elastic constant tensor and isotropic shape of particle, elastic strain also becomes isotropic and satisfies $\varepsilon_{11}^{\text{e}} = \varepsilon_{22}^{\text{e}} = \varepsilon_{33}^{\text{e}}$. Therefore, $a_{297\text{K}}^{\text{strained}}$ can be calculated using any of $\varepsilon_{11}^{\text{e}}$, $\varepsilon_{22}^{\text{e}}$, $\varepsilon_{33}^{\text{e}}$.

The maximum principal stress, denoted as σ_1, often serves as a fracture criterion for brittle materials such as glass. It is determined by solving the eigenvalue problem of the stress tensor σ_{ij}. The eigenvalues of σ_{ij}, corresponding to the principal stresses, are obtained by solving the characteristic equation:

$$\det(\sigma_{ij} - \sigma\delta_{ij}) = 0 \tag{S27}$$

The three real roots of this equation, ordered as $\sigma_1 \geq \sigma_2 \geq \sigma_3$, represent the principal stresses. The largest eigenvalue, σ_1, corresponds to the direction of maximum tensile stress within the material. To assess the risk of glass matrix fracture caused by thermal stress, we calculated the

maximum value, σ_{max}, of the computed σ_1 across the simulation domain and compared it with the mechanical strength of the glass material.

The calculations employed lattice constants a_{297K} and a_{77K} for NH_3 obtained from PBE+D4 calculations. The elastic properties of NH_3 were modeled assuming isotropic elasticity, employing a Poisson's ratio (v) of 0.2 and volume modulus calculated by PBE+D4. To incorporate the pressure dependence of *K*, we fitted the calculated *K* data for different pressure to a second-order polynomial equation of the form $K(\varepsilon_v) = \kappa_\alpha \varepsilon_v^2 + \kappa_\beta \varepsilon_v + \kappa_\gamma$ where ε_v is the volumetric strain $\varepsilon_v(= (1 + \varepsilon_{11})(1 + \varepsilon_{22})(1 + \varepsilon_{33}) - 1)$. The stress and strain arising from the pure expansion of the spherical particle is uniform within the particle.[62] Therefore, the volumetric strain ε_v can be selected at any point within the particle. In the simulation, volumetric strain at the center of the spherical particle was selected as ε_v. For the elastic modulus of B_2O_3, the value measured by Ramos et al. [59] were employed. Table S1 summarizes the parameters used for the phase-field micromechanical elasticity simulation.

Table S1 Parameters used for the phase-field micromechanical elasticity simulation: Lattice constants, a_T, and coefficients for volume modulus of NH_3, κ_l, were determined from the present first principles calculation; elastic constants of B_2O_3, C_{ij}, were values given by Ramos et al.[59] Poisson ratio of NH_3, v, from κ_l.

Parameter	Values	Remarks
$a_T(NH_3)$/nm	a_{297K} = 0.5279; a_{77K} = 0.5100	Present first principles cal.
$C_{ij}(B_2O_3)$/GPa	C_{11} = 24.42; C_{44} = 7.53	Ref. 59
$\kappa_l(NH_3)$/GPa	κ_α = 92.73; κ_β = 13.39; κ_γ = 2.34	Present first principles cal.
$v(NH_3)$	0.2	-

RESULTS

Coefficients for the $C^{\circ}_{p,\mathrm{m}}(\mathrm{NH_3(s)})$ fitting functions

Overstreet and Giauque[35] measured the $C^{\circ}_{p,\mathrm{m}}$ data for solid state ammonia at 15–190 K. Their data were fitted using the Debye–Einstein function given by Equation (2) in the main document. The coefficients for the optimized Debye–Einstein function are listed in Table S2.

Table S2. Coefficients for the optimized Debye–Einstein function fitted with $C^{\circ}_{p,\mathrm{m}}$ data over 15–190 K measured by Overstreet and Giauque.[35]

	Parameters	Coefficients
Low *T* fits	γ / (J K^{-2} mol of compd.$^{-1}$)	1.78959E–02
	B_3 / (J K^{-4} mol of compd.$^{-1}$)	8.86855E–05
	B_5 / (J K^{-6} mol of compd.$^{-1}$)	2.88074E–07
	B_7 / (J K^{-8} mol of compd.$^{-1}$)	–4.29667E–10
	B_9 / (J K^{-10} mol of compd.$^{-1}$)	2.35470E–13
	B_{11}/ (J K^{-12} mol of compd^{-1})	–4.63626E–17
	Range / K	0–33.18

Continued in the next page.

Continued from the previous page.

	Parameters	Coefficients
Mid T fits	C_0 / (J K^{-1} mol of compd.$^{-1}$)	–2.40083E+02
	C_1 / (J K^{-2} mol of compd.$^{-1}$)	2.89626E+01
	C_2 / (J K^{-3} mol of compd.$^{-1}$)	–1.43600E+00
	C_3 / (J K^{-4} mol of compd.$^{-1}$)	3.78861E–02
	C_4 / (J K^{-5} mol of compd.$^{-1}$)	–5.54968E–04
	C_5 / (J K^{-6} mol of compd $^{-1}$)	4.28484E–06
	C_6 / (J K^{-7} mol of compd.$^{-1}$)	–1.36411E–08
	Range / K	33.18–71.58
High T fits	l	3.32700E–01
	θ_D / K	3.03501E+02
	m	3.33780E–01
	θ_{E_1} / K	5.29767E+03
	n	3.33490E–01
	θ_{E_2} / K	2.34982E+04
	A_1 / (J K^{-2} mol of compd.$^{-1}$)	2.44022E–02
	A_2 / (J K^{-4} mol of compd.$^{-1}$)	2.24768E–06
	Range/T	71.58–300

Deviations between experimental and calculated $C^{\circ}_{p,\mathrm{m}}(\mathrm{NH_3(s)})$

The deviations, $u_{\mathrm{fit}}(C^{\circ}_{p,\mathrm{m}})$, between the data calculated from the Debye–Einstein function (Equation (1) and Table S2) and the $C^{\circ}_{p,\mathrm{m}}$ data[35] were 1.34, 1.17, 1.45, and 1.30% at 15.0, 88.70, 93.4, and 103.0 K, respectively. At other temperatures, the $u_{\mathrm{fit}}(C^{\circ}_{p,\mathrm{m}})$ values were within one %.

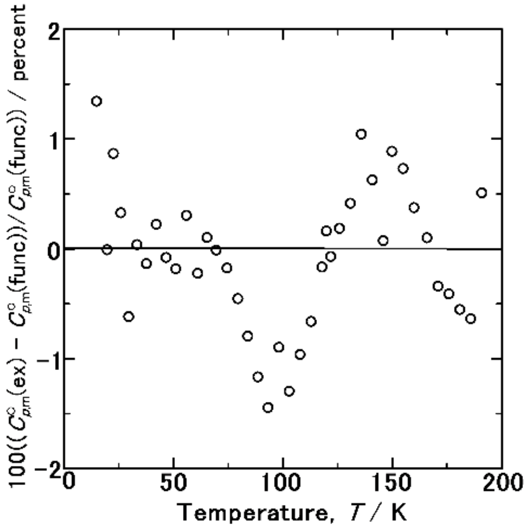

Figure S1. Deviations between experimental $C^{\circ}_{p,\mathrm{m}}$ data for solid ammonia (open circles) at 15–190 K and the results calculated from the fitting functions optimized in the present study (Equations (1)–(3) and Table S2).

Lattice constants and heat capacities from first-principles calculations

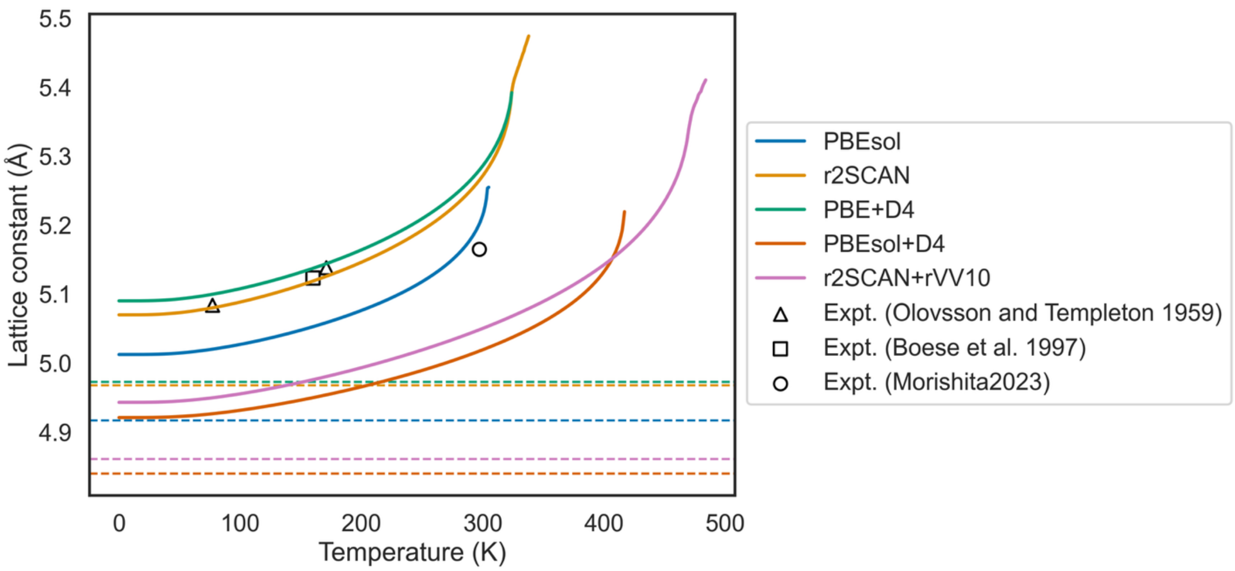

Figure S2. Temperature-dependent lattice constants computed using various exchange-correlation (XC) functionals compared with the experimental data. The solid lines represent the calculated results within the quasiharmonic approximation, whereas the dashed lines indicate the DFT-optimized lattice parameters. The tested XC functionals are PBEsol,[63] r2SCAN,[64] PBE+D4,[54] PBEsol+D4,[54] and r2SCAN+rVV10.[65]

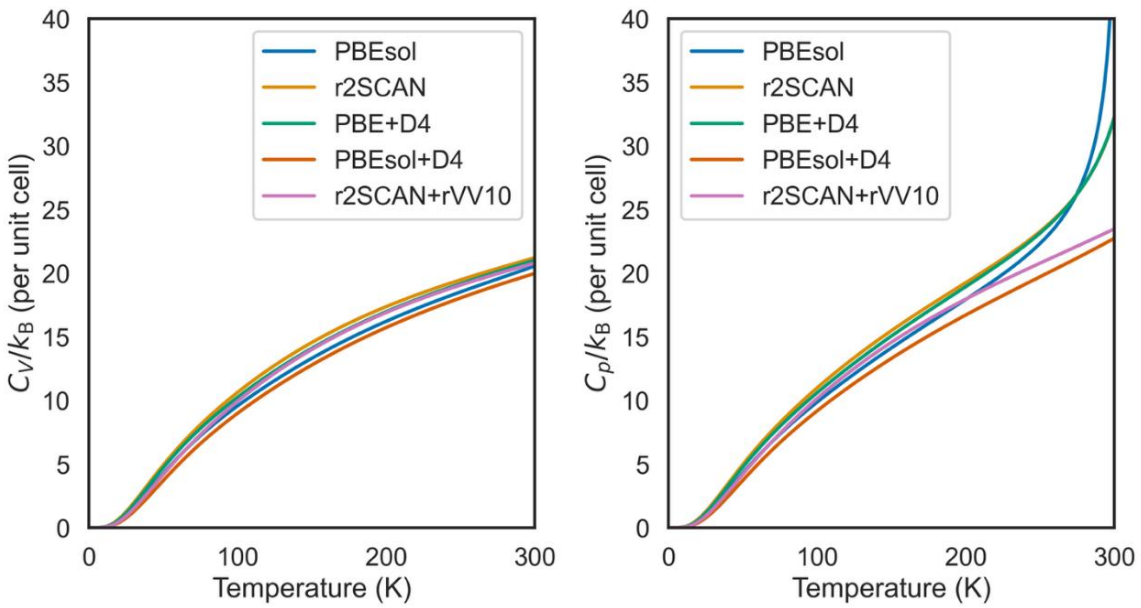

Figure S3. Heat capacity at the constant volume (left) and constant pressure (right) computed using first-principles lattice dynamics within the quasiharmonic approximation.

Thermodynamic data for solid, liquid, and gas phases

The thermodynamic functions for the solid, liquid, and gas phases at equilibrium are summarized in Table S3. The thermodynamic functions for the solid phase, including quasi-equilibrium states above 195.4 K, are summarized in Table S4.

The $\Delta_f G_m^\circ$ value of solid-phase ammonia at 298.15 K was determined by inserting the optimized Debye–Einstein function into Equation (1) in the main text.

Table S3. Thermodynamic values for solid, liquid, and gas phases of ammonia.

T / K	$C^{\circ}_{p,\mathrm{m}}$ / (J K^{-1} (mol compd.)$^{-1}$)	S°_{m} / (J K^{-1} (mol compd.)$^{-1}$)	H°_{m} (T) $-H^{\circ}_{\mathrm{m}}$ (298.15) / (kJ (mol compd.)$^{-1}$)	$\varphi(T)$ / (J K^{-1} (mol compd.)$^{-1}$)	$\Delta_{\mathrm{f}}H^{\circ}_{\mathrm{m}}$ / (kJ (mol compd.)$^{-1}$)	$\Delta_{\mathrm{f}}G^{\circ}_{\mathrm{m}}$ / (kJ (mol compd.)$^{-1}$)
Solid						
0	0	0	–39.157	Infinite	-	-
20	1.550	0.711	–39.148	0.713	-	-
50	11.130	5.626	–38.965	5.627	-	-
100	26.495	18.288	–38.007	18.288	–72.860	–51.611
150	38.527	31.329	–36.379	31.329	–73.980	–41.204
195.4	51.049	43.027	–34.358	43.027	–74.460	–30.661
Liquid*1						
195.4	73.339	71.968	–28.703	71.968	–68.804	–30.661
200	73.614	73.678	–28.365	73.678	–68.714	–29.671
239.7	75.981	87.215	–25.395	87.215	–68.006	–22.263
Gas*2						
239.7	34.515	184.632	–2.044	184.632	–44.655	–22.263
250	34.338	186.079	–1.690	186.079	–44.888	–21.301
298.15	34.547	192.258	0	192.258	–45.940	–16.319
300	35.678	192.479	0.066	192.479	–45.980	–16.136

*1 $\Delta_{\mathrm{f}}G^{\circ}_{\mathrm{m}}(\mathrm{l})/(\mathrm{kJ\ (mol\ of\ compd.)^{-1}}) = -68.139 + 0.1918T$

*2 $\Delta_{\mathrm{f}}G^{\circ}_{\mathrm{m}}(\mathrm{l})/(\mathrm{kJ\ (mol\ of\ compd.)^{-1}}) = -46.819 + 0.1023T$

Table S4. Thermodynamic values for solid phase ammonia.

T / K	$C^{\circ}_{p,\mathrm{m}}$ / (J K^{-1} (mol compd.)$^{-1}$)	S°_{m} / (J K^{-1} (mol compd.)$^{-1}$)	$H^{\circ}_{\mathrm{m}}(T)-H^{\circ}_{\mathrm{m}}(298.15)$ / (kJ (mol compd.)$^{-1}$)	$\varphi(T)$ / (J K^{-1} (mol compd.)$^{-1}$)	$\Delta_{\mathrm{f}}H^{\circ}_{\mathrm{m}}$ / (kJ (mol compd.)$^{-1}$)	$\Delta_{\mathrm{f}}G^{\circ}_{\mathrm{m}}$ / (kJ (mol compd.)$^{-1}$)
0	0	0	–12.195	Infinite	-	-
20	1.550	0.711	–12.186	0.712	-	-
50	11.130	5.626	–12.003	5.626	-	-
100	26.495	18.288	–11.046	18.288	–72.860	–51.611
150	38.527	31.329	–9.417	31.329	–73.980	–41.204
195.4	51.049	43.027	–7.396	43.027	–74.460	–30.661
200	52.528	44.232	–7.158	44.232	–74.469	–29.537
239.7	67.484	54.993	–4.79	54.993	–74.362	–20.895
250	72.092	57.928	–4.071	57.928	–74.230	–18.606
298.15	98.384	72.753	0	72.753	–72.902	–7.650
300	99.564	73.365	0.183	73.365	–72.825	–7.246

Hydrostatic stress for phase transitions at 298.15 K

Figs. S4(a), (b) and (c) shows the NH_3 article placed in the B_2O_3 matrix and calculated hydrostatic and maximum principal stress field, respectively. The NH_3 particles exhibited a uniform σ_{hydro} of 0.292 GPa. The maximum principal stress was compressive within the NH_3 particle, while the B_2O_3 matrix experienced tensile stresses, reaching a maximum value of 0.122

GPa at the particle/matrix interface. These results demonstrate that the NH_3 particles are subjected to sub-GPa thermal stresses, while B_2O_3 matrix experiences substantial tensile forces.

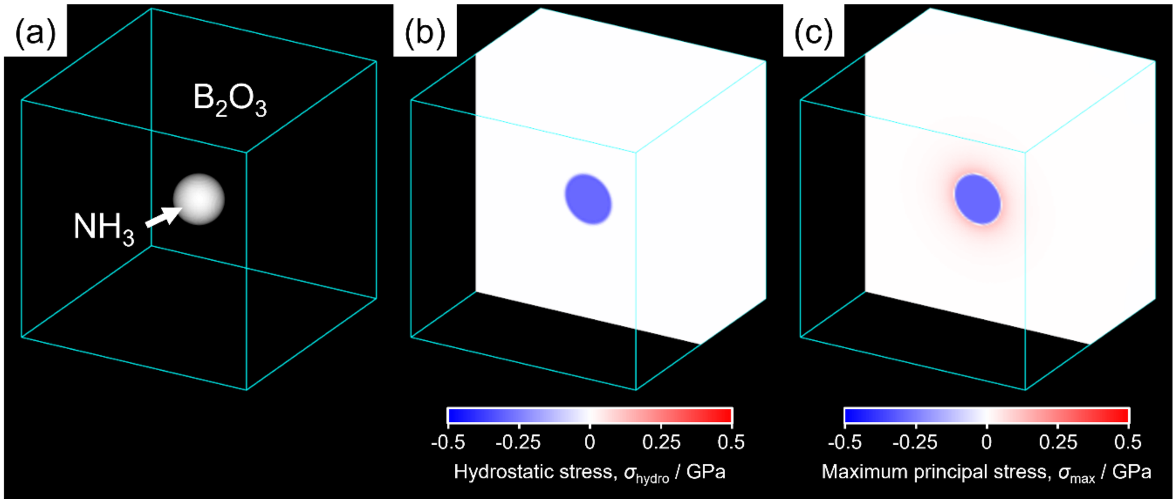

Figure S4. NH_3 article placed in the B_2O_3 matrix (a), and hydrostatic stress field (b) and maximum principal stress field (c) calculated by phase-field micromechanical elasticity simulation. Positive value (red) indicates tensile stress and negative value (blue) indicates compressive stress.

Table S5. Estimated hydrostatic stress, σ_{hydro}, experienced by the NH_3 particles and maximum principal stress, σ_{max}, acting on glass matrix (GM).

297 K	GPa	Remarks
$\sigma_{\mathrm{hydro}}(NH_3(s))$	0.292	Compressive stress
$\sigma_{\mathrm{max}}(\mathrm{GM})$	0.122	Tensile stress
